# Effects of Supplementation with Oregano Essential Oil during Late Gestation and Lactation on Serum Metabolites, Antioxidant Capacity and Fecal Microbiota of Sows

**DOI:** 10.3390/ani14050753

**Published:** 2024-02-28

**Authors:** Yuanyi Zhang, Yuhang Deng, Yubin Hao, Jianmin Fang, Jie Feng

**Affiliations:** 1 Key Laboratory of Animal Nutrition and Feed of Zhejiang Province, College of Animal Sciences, Zhejiang University, Hangzhou 310058, China; york.zhang@zju.edu.cn (Y.Z.);; 2 Keqiao Animal Husbandry and Veterinary Research Institute, Shaoxing 312030, China

**Keywords:** oregano essential oil, sow, serum metabolites, antioxidant capacity, fecal microbiota

## Abstract

**Simple Summary:**

It is a big challenge to maintain a healthy state in sows during late gestation and lactation, and it directly impacts the performance of both sows and their offspring. Essential oil, consisting of multiple active compounds, is regarded as a promising feed additive. In this study, maternal diets were supplemented with oregano essential oil during late gestation and lactation to investigate its potential effects. We collected and analyzed serum and fecal samples from sows to evaluate performance, serum metabolites, antioxidant capacity and fecal microbiota, aiming to provide valuable insights into the utilization of oregano essential oil in animal production.

**Abstract:**

A total of 20 healthy white × landrace sows were evenly and randomly divided into two groups, and fed basal diets unsupplemented or supplemented with 500 g/t Meriden-Stim^®^ from day 100 of gestation until day 21 of lactation. Serum and fecal samples were collected from the sows on the final day for subsequent analysis. Compared to the control group, there were no significant differences in the sows’ performances; however, an increase was observed in the piglets’ weight at weaning (*p* = 0.08). Moreover, oregano essential oil (OEO) significantly reduced the levels of urea (UREA) (*p* < 0.01), total cholesterol (TC) (*p* < 0.05), low-density lipoprotein (LDL-C) (*p* < 0.05) and alanine aminotransferase (ALT) (*p* < 0.05) in serum. In terms of antioxidant indexes in serum, the catalase (CAT) and glutathione (GSH) levels showed significant increases (*p* < 0.05) while the malondialdehyde (MDA) level exhibited a decrease tendency (*p* = 0.09). 16S rRNA analysis identified the specific bacteria taxa in feces. OEO significantly decreased the relative abundance of Proteobacteria and Actinobacteria at the phylum level (*p* < 0.05). At the genus level, OEO significantly increased the relative abundance of *Lactobacillus* and *Prevotellaceae UCG 003* and *UCG 005*, while decreasing that of *Escherichia-Shigella* (*p* < 0.05). Taken together, OEO supplementation in maternal diets during late gestation and lactation improved serum metabolites, antioxidant capacity and regulated the intestinal-flora balance of sows, thereby tending to increase the piglets’ weight at weaning.

## 1. Introduction

During the reproductive cycle, sows undergo placentation, fetal development, parturition and lactation with an elevated metabolic level to meet the nutritional demands of their offspring [1,2]. Additionally, their physiological condition changes significantly in a comprehensive way involving indicators related to hepatorenal and cardiac function [3,4,5]. After delivery, the sows mobilize their body reserves for milk secretion as ad libitum feed intake is insufficient to fully support the growth of piglets [6]; consequently, substantial protein and fat will be lost during this period [7,8], and such intensified metabolic levels may induce oxidative stress [9]. Reactive oxygen species (ROS), including superoxide and hydrogen peroxide produced by both the placenta and maternal tissues, accumulate significantly during this period [10]. A disruption of the balance between ROS production and antioxidant capacity leads to oxidative stress, which negatively affects reproductive performance such as decreased total litter size, live litter size and litter weight gain [11]. Moreover, reduced feed intake during lactation will result in a prolonged negative energy balance and greater loss of body condition, as well as reduced milk production [12].

The gut microbiota play a crucial role in regulating various metabolic processes in the host, including energy homeostasis, glucose metabolism and lipid metabolism [13]. The composition and function of gut microbiota are affected by the host genetics, dietary factors [14], antibiotics usage [15], etc. Moreover, gut microbiota undergo alterations during different physiological stages, particularly gestation and lactation [16]. Compared to the early gestation, there is a significant increase in Proteobacteria and Actinobacteria in the sow gut at the late stage, which has clear characteristics associated with increased risk of inflammation and energy loss [17]. Numerous studies have demonstrated that maternal gut microbiota can be transmitted to the fetus through the placenta or milk [18], highlighting their essential role in both maternal and fetal performance [19].

Oregano essential oil (OEO) is isolated from *Origanum vulgare* L. through steam distillation. Chemical analysis has shown that carvacrol and thymol are its major constituents [20]. It has been widely used as a feed additive and demonstrated efficacy in antimicrobial and antioxidant activities [21]. Furthermore, several studies have confirmed that OEO intervention can enhance hepatorenal functions in fish species [22] and influence lipid profiles in humans [23]. However, limited research exists on the effects of OEO supplementation during late gestation and lactation on pregnant sows. In this study, sows were fed diets unsupplemented or supplemented with OEO from day 100 of gestation until day 21 of lactation. The reproductive performance, serum parameters, antioxidant capacity and fecal microbiota of the sows and the growth performance of the piglets were determined.

## 2. Materials and Methods

### 2.1. Reagents

The product Meriden-Stim^®^, obtained from Anpario Ltd. (Worksop, UK), consists of 5% OEO (extracted from *Origanum vulgare subsp. hirtum*) and 95% natural feed grade inert carrier. Major constituents of OEO are carvacrol (≥55.00%) and thymol (≥1.50%).

### 2.2. Animals and Treatment

Twenty large white × landrace sows [average body weight (BW), 299.07 ± 17.79 kg; parity, 3.75 ± 0.79] on day 100 of gestation were divided into two groups, each comprising ten sows, based on similar body weight and parity.

The basal diet (Table 1) was formulated based on the nutritional requirements for pregnant and lactating sows as per the NRC 2012 guidelines. In addition to the basal diet, the experimental group was supplemented with 500 g/t Meriden-Stim^®^.

The experimental period lasted from day 100 of gestation until day 21 of lactation. Sows were housed individually in pens (2.4 m × 1.8 m) in a farrowing house during the experiment. The temperature in the farrowing room was consistently maintained in a range of 22–28 °C, and the room remained illuminated throughout the entire period.

Sows were restricted to a 3–3.5 kg daily diet during late gestation and had ad libitum access to both feed and water throughout the entire lactation period. Feed was supplied three times a day (7:00, 15:00 and 20:00). Piglets had free access to sow’s milk and received routine management practices. Piglets were supplied with creep feed on day 7 postpartum and weaned on day 21 postpartum. All sows were delivered within 3 days.

### 2.3. Sow and Piglet Performance

BW of sows was measured individually within 24 h of farrowing and at weaning. Backfat thickness was measured the day prior to parturition and at weaning. Backfat thickness at 65 mm on each side of the dorsal midline at the last rib was measured using ultrasound (Lean-Meater, Renco Corporation, Golden Valley, MN, USA). At farrowing, the numbers of total piglets born and piglets born alive were recorded. The piglets were cross-fostered within dietary treatment groups by 48 h after farrowing to adjust the litter size. The number of piglets per sow ranged from 11 to 14 piglets. At weaning, the numbers of weaned piglets were recorded.

The litter performance of sows was assessed based on birth and weaning survival rates of piglets, weight loss and backfat loss of sows and weaning to estrus interval (WEI). Feed intakes during gestation and lactation were recorded each morning by weighing daily feed refusals. Total litter weights per sows on birth day and days 7, 14 and 21 after birth were measured.

### 2.4. Sample Collection

Blood samples were collected from all sows on day 21 of lactation via the ear vein and transferred into 5 mL tubes. The serum was separated by centrifugation at 3000× rpm for 10 min at 4 °C and subsequently stored in 1.5 mL tube aliquots at −80 °C. Fecal samples were collected immediately from five sows in each group after excretion in the same day and stored in 50 mL tubes at −80 °C.

### 2.5. Determination of Serum Biochemical Parameters

The Affiliated Hospital of Hangzhou Normal University (Hangzhou, China) was entrusted with the task of determining partial serum biochemical parameters, including urea (UREA) (enzymic method), creatinine (CR) (sarcosine oxidase method), total cholesterol (TC) (enzymic method), triglycerides (TG) (GPO-POD method), high-density lipoprotein (HDL-C) (direct method), low-density lipoprotein (LDL-C) (direct method), aspartate aminotransferase (ALT) (enzymic method) and alanine aminotransferase (AST) (MDH method). All the above indexes were measured by an automatic biochemical analyzer.

The levels of superoxide dismutase (SOD) (A001-3, WST-1 method), catalase (CAT) (A007-1-1, ammonium molybdate spectrometric method), malondialdehyde (MDA) (A003-1, TBA method) and glutathione (GSH) (A006-2-1, DTNB method) in serum were quantified using assay kits (Jiancheng, Nanjing, China).

### 2.6. 16S rRNA-Based Microbiota Analysis

The task of performing 16S rRNA amplicon sequencing to analyze the microbiomes for fecal samples collected from five sows in each group was assigned to Novogene Technology Co., Ltd. (Beijing, China).

The cetyltrimethylammonium bromide (CTAB)/SDS method was employed to extract the genomic DNA from the samples. The 16S V3-V4 sequenced region was selected for polymerase-chain-reaction (PCR) amplification. The library was constructed using a TruSeq^®^ DNA PCR-Free Sample Preparation Kit and then checked with Qubit and Q-PCR for quantification. After library qualification, on-machine sequencing (PE250) was performed on the Illumina NovaSeq6000 to obtain off-machine data. Then, the obtained data underwent splicing and quality control processes to generate clean tags. Chimera filtering was conducted to obtain effective tags suitable for subsequent analysis. The UPARSE algorithm (Version 7.0.1001) was employed to cluster all samples’ effective tags into operational taxonomic units (OTUs) with 97% identity, followed by the species annotation of OTU sequences.

The top 10 species with the largest abundance at each taxonomic level were selected for each sample or group, and a column accumulation chart illustrating the relative abundance of species was generated. Alpha diversity analysis and beta diversity analysis were conducted using the Qiime2 software (Version 202202). Then, the *T*-test, Wilcoxon rank-sum test and Tukey test were used through R software (Version 4.0.3) to analyze the intergroup variation in species diversity and determine if any significant differences in mean gender existed. Linear discriminant analysis (LDA) effect size (LEfSe) analysis was conducted using the LEfSe software (Version 1.1.01), with a filter value of LDA Score set to 4.0. Species with significant differences between groups were subjected to a between-group *T*-test using R software and subsequently plotted.

### 2.7. Statistical Analysis

Individual sows and total piglets in a litter were considered as independent experimental units for analyzing the litter performances of sows and the growth performances of piglets. All data are presented as mean ± standard deviation (SD). Means were compared using a *T*-test; differences between treatment means were significant at *p* < 0.05 and trends were identified, when *p* ≥ 0.05, but <0.10. All statistical analyses were performed using the SPSS 26.0 software and plotted with the Graphpad Prism 8.0 software.

## 3. Results

### 3.1. Sow Performance

The results for sows performance are shown in Table 2. The BW, backfat thickness, average daily feed intake (ADFI) and WEI were not affected significantly by OEO supplementation (*p* > 0.05).

### 3.2. Piglet Performance

The results for the piglets are shown in Table 3. There were no significant differences in the number of total piglets born, live-born and weaned (weaning survival rate) (*p* > 0.05). There was a trend of increase in the weaning BW (0.05 ≤ *p* < 0.01) compared to the control group.

### 3.3. Serum Biochemical Parameters and Antioxidant Capacity of Sows

The results for the serum biochemical parameters of the sows are shown in Table 4. Compared to the control group, the blood urea nitrogen (BUN)/CR (*p* < 0.01), levels of UREA (*p* < 0.01), TC (*p* < 0.05), LDL-C (*p* < 0.05) and AST (*p* < 0.05) decreased.

**Figure 1:**
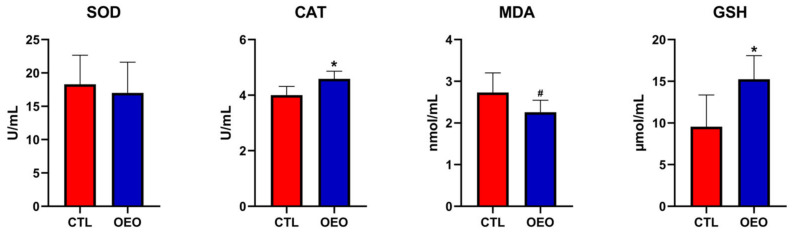
Effects of OEO supplementation during late gestation and lactation of sows on antioxidant capacity. SOD, superoxide dismutase; CAT, catalase; MDA, malondialdehyde; GSH, glutathione. *, *p* < 0.05; #, 0.05 ≤ *p*< 0.10.

The results for the antioxidant capacity are shown in Figure 1. The CAT (*p* < 0.05) and GSH (*p* < 0.05) levels increased while the MDA (0.05 ≤ *p*< 0.01) level had a tendency to decrease.

### 3.4. Composition and Differences of Fecal Microbiota

**Figure 2:**
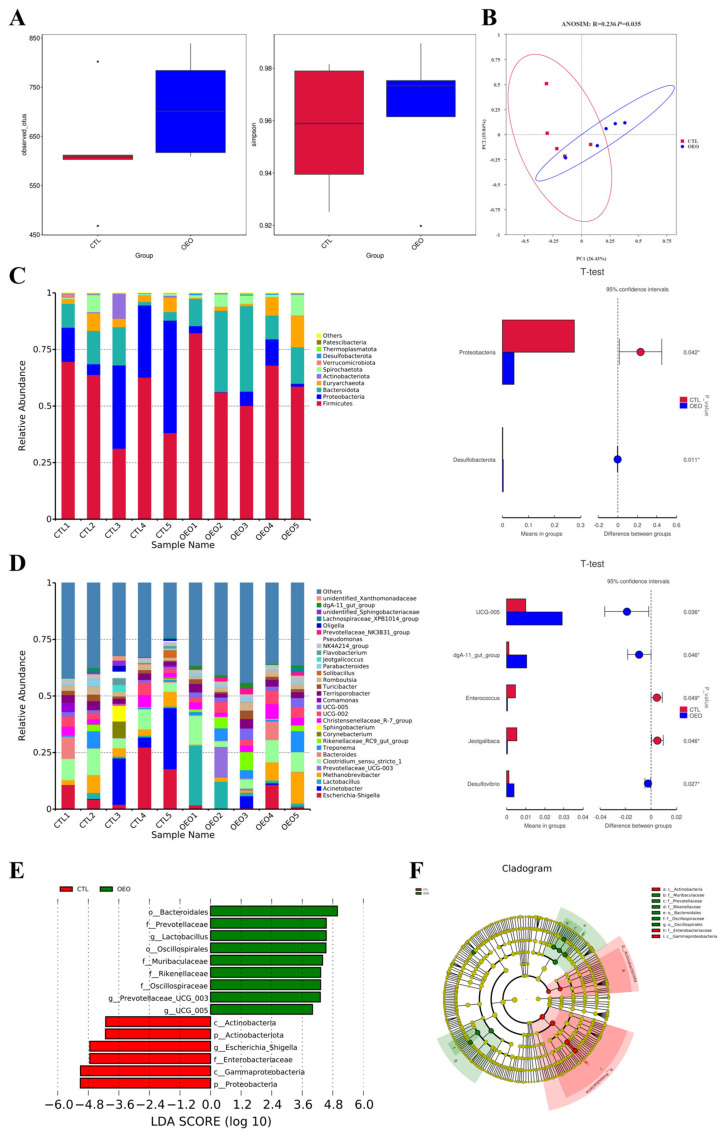
Effects of OEO in fecal microbiota of sows on day 21 of lactation: (**A**) Alpha diversity analysis; (**B**) beta diversity analysis based on Bray-Curtis; (**C**) top 10 relative abundance of fecal microbiota at the phylum level; (**D**) top 30 relative abundance of fecal microbiota at the genus level; (**E**) linear discriminant analysis (LDA) score histogram shows taxonomic biomarkers [the LDA score (log10) > 4]; (**F**) LEfSe evolutionary cladogram showing the phylogenetic distribution of the fecal microbiota. Sows were regarded as the experimental units, *n* = 5 for each group. *, *p* < 0.05.

The results of the 16S rRNA analysis are presented in Figure 1, showcasing the findings from various analyses. The alpha diversity analysis (Figure 2A) revealed no significant differences in the observed species and Simpson index between the two groups (*p* > 0.05). The principal coordinate analysis (PCoA) based on the Bray-Curtis distance was employed to evaluate the fecal microbiota composition of the control and OEO groups (Figure 2B), demonstrating a significant change in fecal microbiota composition (*p* < 0.05).

The top 10 relative abundances of bacteria at the phylum level of all samples are presented in Figure 2C and the top 30 at the genus level are presented in Figure 2D. Compared to the control group, OEO decreased the relative abundance of the Proteobacteria while it increased that of the Desulfobacterota at the phylum level. At the genus level, the OEO increased the relative abundance of the *UCG-005*, *dgA-11 gut group* and *Desulfovibrio* while decreasing that of *Enterococcus* and *Jeotgalibaca* significantly (*p* < 0.05).

By employing LEfSe analysis, we assessed the fecal microbial differential species to discern specific bacterial taxa in both the control and OEO groups. Figure 2E illustrates the species with significant differences, represented by the LDA score > 4.0, reflecting the degree of influence of species with significant differences between groups. At the phylum level, OEO significantly decreased the relative abundance of Proteobacteria and Actinobacteria (*p* < 0.05). At the genus level, OEO significantly increased the relative abundance of *Lactobacillus* and *Prevotellaceae UCG 003* and *UCG 005*, while decreasing that of *Escherichia-Shigella* (*p* < 0.05).

## 4. Discussion

Previous studies investigated the effects of OEO supplementation on parameters such as the growth performance, production performance, health state and microbiota composition of animals [22,24]. However, limited attention has been given to its effects on sows, especially during late gestation and lactation. Therefore, this study was conducted and observed few differences in sow performance and an increase tendency in piglet weight at weaning. We hypothesize that the favorable piglet performance may be attributed to two main active compounds in OEO, thymol and carvacrol, both of which have been reported to possess antimicrobial, antioxidant, anticarcinogenic, anti-inflammatory and antispasmodic activities, as well as their potential role as growth enhancers and immunomodulators [25,26].

Serum parameters are valuable indicators of the health and wellbeing of sows. The serum parameters observed in this study were all within the normal physiological range for sows [27,28]. The enzymes AST and ALT participate in transamination reactions by facilitating the transfer of amino groups from aspartate or alanine to ketoglutaric acid, resulting in the formation of oxaloacetic acid or pyruvate. These reactions play a crucial role in the gluconeogenesis process. Elevated levels of AST, ATL or AST/ALT ratio in serum often suggest increased liver cell permeability and liver damage [29]. Among these indexes, the AST/ALT ratio is widely accepted and holds significant clinical relevance due to its demonstrated associations with various diseases and even mortality [30]. Our study observed lower levels of these indexes in the OEO group. Similar reductions have been reported in fish studies [22]. A review has revealed that numerous essential oils, containing cineol, carvacrol or thymol as major compounds, exhibited hepatoprotective and antioxidant functions simultaneously across various studies [31]. Therefore, it is plausible that the abundant antioxidant compounds present in OEO contribute to its hepatoprotective effects.

Furthermore, the nephroprotective potential of OEO was assessed by measuring the UREA and CR levels; both decreased, and the UREA level decreased significantly compared to the control group. Additionally, our calculations revealed a significant reduction in the BUN/CR ratio, indicating that OEO has the possibility to enhance the utilization of nitrogenous nutrients. Notably, this protective effect was more significant under impaired conditions [26]. Thymol has demonstrated similar efficacy in murine models [32]; however, further investigation is required to elucidate the underlying mechanisms, as limited studies have reported on essential oil’s or its components’ (thymol or carvacrol) possession of this function, indicating that the specific active compound remains unidentified.

Our study revealed changes to lipid metabolism, characterized by a significant reduction in TC and LDL-C levels, with no significant differences observed in TG and HDL-C levels. During pregnancy, there is a physiological increase in TC, TG and LDL-C levels and a decrease in the HDL-C level, due to increased insulin resistance, progesterone, estrogens and placental lactogen [33]. These changes are strongly associated with an increased risk of atherosclerosis development and even coronary heart disease [34]. Thymol and carvacrol have been reported to exhibit beneficial effects on serum lipid metabolism [35,36]. The potential hypocholesterolemic effects of thymol and carvacrol may be ascribed to the inhibition of 3-hydroxy-3-methylglutaryl coenzyme A reductase, the rate-limiting enzyme in cholesterol synthesis [37]. However, a study on spontaneously hypertensive rats reported that exclusive treatment with carvacrol did not yield any improvements in TC, non-HDL cholesterol, HDL-C levels or LDL-C levels [38]. Therefore, it can be inferred that the antihyperlipidemic effect may arise from the synergistic interplay of multiple compounds present in OEO. The TG/HDL-C ratio has been recognized as a risk marker for metabolic syndrome and cardiovascular disease, based on an extensive analysis of human medical data, demonstrating that a higher ratio is closely associated with increased health risks [39]. Our calculations revealed no significant differences in this ratio, further supporting the findings in the present study that OEO mainly improves serum lipid metabolism by reducing TC and LDL-C levels.

During late gestation and lactation, sows undergo substantial metabolic changes [40]. Increased metabolic burdens cause elevated systemic oxidative stress during these specific periods [41], and the process involves a variety of enzymatic and non-enzymatic mechanisms. The main non-enzymatic endogenous antioxidant, GSH, can directly protect cells against free radicals and pro-oxidants and act as a cofactor for antioxidants and detoxification enzymes. In our study, OEO supplementation significantly increased the concentration of GSH in serum on day 21 of lactation, which might indicate that the sows suffered from great oxidative stress during lactation and benefited from the effects of OEO. The product of lipid peroxidation, MDA, is a widely accepted biomarker of oxidative stress [42]. The accumulation of MDA damages the sows’ body conditions and, subsequently, the piglets’ performances. A lower level of MDA was found in our study. CAT and SOD are two types of antioxidant enzymes, the former responsible for clearing hydrogen peroxide and the latter for superoxide. OEO supplementation increased the CAT level. These positive effects of OEO are most likely attributable to its major constituents carvacrol and thymol, both of which have been reported to scavenge superoxide radicals and hydrogen peroxide [43,44]. An in vitro study concluded that treatment of IPEC-J2 cells with OEO enhanced the SOD and GSH expression through activation of the Nrf2/ARE pathway, which may be pivotal to its antioxidative action against H2O2-induced cell damage [45].

The fecal microbiota is closely related to the host health and involved in various host metabolic processes. Our study has found that OEO supplementation did not affect the richness and evenness of the microbiota between the two groups, but altered the composition of the microbiota significantly. Subsequently, we distinguished the key bacteria taxa between the control and OEO groups to pinpoint the differences. An increased abundance of Proteobacteria and Actinobacteria are linked to elevated risk inflammation and energy loss [17]. Furthermore, an increased prevalence of Proteobacteria is a marker for an unstable microbial community and a potential diagnostic criterion for disease [46]. At the phylum level, there was a significant decrease in their relative abundance in the OEO group, suggesting that sows may conserve more energy for higher quantity and quality milk secretion.

*Escherichia-Shigella*, a genus belonging to the Proteobacteria phylum, is closely associated with gut dysbiosis [46] and exhibited a decrease in abundance in the OEO group. As a zoonotic pathogen, *E. coli O157:H7* is the most important serotype of Shiga toxin-producing *E. coli*. A study found that *E. coli O157:H7* induced significant elevation in serum creatinine and urea levels, and *Thymus vulgaris* essential oil could alleviate the hepatorenal dysfunction [47], aligning with our study’s findings on serum and microbiota data. Another investigation has shown how carvacrol inhibited flagellin production and, therefore, flagellar development, leaving *E. coli O157:H7* immobile due to a heat shock of protein [48]. Additionally, various essential oils have been reported to exhibit distinct antimicrobial effects and numerous theories suggest that the mechanism of action is mostly related to their hydrophobic properties, which interact with the cell membrane [49].

Prevotella bacteria encode a large number of carbohydrate-degrading enzymes and are known producers of short-chain fatty acids, which can also participate in the long-term regulation of energy metabolism [50]. An increase in the abundance of *Prevotellaceae UCG 003* and *UCG 005* was observed, potentially beneficial to satiety in pregnant sows. It is noteworthy that the relationship between Prevotella and nutrition should receive more research focus since multiple convincing studies have demonstrated an association between Prevotella and dietary patterns; however, such studies often report conflicting results [51].

An increased abundance of *Lactobacillus* was also observed, beneficial for maintaining intestinal flora balance and inhibiting the growth of harmful bacteria. Previous studies have shown that *Lactobacillus* compete with intestinal pathogenic bacteria for binding sites in the mucosal layer, thereby suppressing bacterial infection [52]. An adhesion assay revealed a 59.71% reduction in *E. coli O157:H7* adherence to collagen when using purified collagen-binding protein [53]. These findings provide a possible explanation for the changes in abundance of *Lactobacillus* and *Escherichia-Shigella* observed in our study. The bacteriocin and organic acid produced by *Lactobacillus* also possess pathogenic bacterium antagonist effects. A study reported that the oral administration of *Lactobacillus plantarum* CAM6 in sows from the last third of gestation until weaning decreased the number of deaths of the piglets before weaning and those suffering from the diarrheal syndrome, and improved the weight-gain performance of their offspring weekly [54], which partially supported a link between increased piglet weight at weaning and the higher abundance of *Lactobacillus* observed in our study.

## 5. Conclusions

Our study found that OEO supplementation in maternal diets during late gestation and lactation improved serum metabolites, as evidenced by decreased levels of UREA, TC, TG and AST in the serum and the antioxidant capacity of the sows. Moreover, the ability to regulate the intestinal flora balance of sows, enriching beneficial bacterium like *Lactobacillus* and suppressing harmful ones like *Escherichia-Shigella*, was demonstrated. These findings contribute to the observed increase in piglet weight at weaning.

## Figures and Tables

**Table 1:** Composition and chemical analysis of the late gestation and lactation diets.

Item	Ratio (%)
Corn	44.30
Beet pulp	3.00
Brown rice	10.00
Wheat flour	10.00
Limestone	0.90
Fermented soybean meal	3.00
Extruded soybean	12.00
Soybean meal	11.00
Soybean oil	1.80
Premix ^1^	4.00
Crude protein	17.06
Ether extract	6.09
Crude fiber	2.56
Calcium	1.00
Total phosphorus	0.67
Lysine	1.10
Methionine	0.31
Cystine	0.30
Threonine	0.68
Tryptophan	0.21
Digestible energy ^2^ (Mcal/kg)	3.46

^1^ The premix provides the following in a kilogram of feed: Fe, 80 mg; Cu, 5 mg; Zn, 50 mg; Mn, 20 mg; I, 0.14 mg; Se, 0.30 mg; Vitamin A, 10000 IU; Vitamin D3, 2160 IU; Vitamin E, 15 mg; Vitamin K3, 1.7 mg; Vitamin B1, 2 mg; Vitamin B2, 5 mg; Vitamin B6, 1.8 mg; Vitamin B12, 0.02 mg; Nicotinic, 20 mg; calcium pantothenate, 10 mg; folic acid, 1 mg; and biotin, 0.08 mg. ^2^ Digestible energy was calculated according to the feedstuff composition and corresponding digestive energy.

**Table 2:** Effects of OEO supplementation during late gestation and lactation of sows on sows’ performance.

Item	CTL	OEO	*p* Value
Sow BW (kg)			
Parturition	271.1 ± 14.6	288.5 ± 20.0	0.08
Weaning	225.0 ± 18.1	241.4 ± 27.5	0.20
Loss	46.1 ± 8.0	47.1 ± 16.9	0.88
Sow backfat thickness (mm)			
Day 113	18.8 ± 1.5	18.9 ± 1.4	0.87
Weaning	14.7 ± 1.7	15.3 ± 1.0	0.33
Loss	4.1 ± 0.6	3.6 ± 1.5	0.32
Lactation ADFI (kg)	5.06 ± 0.59	4.90 ± 0.48	0.75
WEI (d)	5.38 ± 0.74	5.22 ± 0.44	0.61

BW, body weight; ADFI, average daily feed intake; WEI, weaning-to-estrus interval.

**Table 3:** Effects of OEO supplementation during late gestation and lactation of sows on piglets’ performance.

Item	CTL	OEO	*p* Value
Litter Size			
Total born	15.29 ± 1.98	16.14 ± 3.48	0.58
Born alive	15.00 ± 2.00	15.29 ± 2.63	0.82
After cross-foster	13.57 ± 0.53	13.71 ± 0.76	0.69
Pigs weaned	13.00 ± 1.00	13.14 ± 1.07	0.80
Weaning survival rate (%)	95.76 ± 5.96	95.84 ± 5.67	0.98
Piglet mean BW (kg)			
At birth	1.55 ± 0.09	1.62 ± 0.22	0.51
After cross-foster	1.60 ± 0.07	1.63 ± 0.21	0.75
At day 7	2.91 ± 0.26	2.91 ± 0.15	0.97
At day 14	4.62 ± 0.34	4.75 ± 0.20	0.45
At day 21	6.31 ± 0.29	6.71 ± 0.41	0.08

BW, body weight.

**Table 4:** Effects of OEO supplementation during late gestation and lactation of sows on serum biochemical parameters.

Item	CTL	OEO	*p* Value
UREA (mmol/L)	6.86 ± 0.43	5.06 ± 0.62	<0.01
CR (μmol/L)	195.65 ± 21.08	178.80 ± 22.08	0.21
BUN/CR	35.19 ± 1.77	28.52 ± 3.94	<0.01
TC (mmol/L)	2.29 ± 0.37	1.87 ± 0.21	0.04
TG (mmol/L)	0.38 ± 0.06	0.35 ± 0.05	0.36
HDL-C (mmol/L)	0.93 ± 0.17	0.82 ± 0.11	0.25
LDL-C (mmol/L)	1.16 ± 0.15	0.94 ± 0.14	0.02
TG/HDL-C	0.37 ± 0.10	0.42 ± 0.08	0.24
AST (U/L)	59.00 ± 16.16	41.86 ± 10.61	0.04
ALT (U/L)	37.67 ± 12.16	32.86 ± 8.99	0.43
AST/ALT	1.64 ± 0.48	1.29 ± 0.19	0.10

UREA, urea; CR, creatinine; BUN, blood urea nitrogen; TC, total cholesterol; TG, triglyceride; HDL-C, high-density lipoprotein; LDL-C, low-density lipoprotein; ALT, aspartate aminotransferase; AST, alanine aminotransferase.

## Data Availability

Data are contained within the article.

## References

[1] Grahofer A., Plush K. (2023). Lactation in Swine: Review Article. Anim. Front..

[2] Almeida F.R.C.L., Dias A.L.N.A. (2022). Pregnancy in Pigs: The Journey of an Early Life. Domest. Anim. Endocrinol..

[3] Zhu Q., Xie P., Li H., Blachier F., Yin Y., Kong X. (2021). Dynamic Changes of Metabolite Profiles in Maternal Biofluids During Gestation Period in Huanjiang Mini-Pigs. Front. Vet. Sci..

[4] Peng X., Yan C., Hu L., Huang Y., Fang Z., Lin Y., Xu S., Feng B., Li J., Zhuo Y. (2020). Live Yeast Supplementation during Late Gestation and Lactation Affects Reproductive Performance, Colostrum and Milk Composition, Blood Biochemical and Immunological Parameters of Sows. Anim. Nutr..

[5] Sapkota A., Marchant-Forde J.N., Richert B.T., Lay D.C. (2016). Including Dietary Fiber and Resistant Starch to Increase Satiety and Reduce Aggression in Gestating Sows. J. Anim. Sci..

[6] Tokach M.D., Menegat M.B., Gourley K.M., Goodband R.D. (2019). Review: Nutrient Requirements of the Modern High-Producing Lactating Sow, with an Emphasis on Amino Acid Requirements. Animal.

[7] Ye H., Langendijk P., Jaworski N.W., Wu Y., Bai Y., Lu D., Page G., Kemp B., Han D., Soede N.M. (2022). Protein Digestion Kinetics Influence Maternal Protein Loss, Litter Growth, and Nitrogen Utilization in Lactating Sows. Front. Nutr..

[8] Tummaruk P., Sumransap P., Jiebna N. (2014). Fat and Whey Supplementation Influence Milk Composition, Backfat Loss, and Reproductive Performance in Lactating Sows. Trop. Anim. Health Prod..

[9] Berchieri-Ronchi C.B., Kim S.W., Zhao Y., Correa C.R., Yeum K.-J., Ferreira A.L.A. (2011). Oxidative Stress Status of Highly Prolific Sows during Gestation and Lactation. Animal.

[10] Hussain T., Murtaza G., Metwally E., Kalhoro D.H., Kalhoro M.S., Rahu B.A., Sahito R.G.A., Yin Y., Yang H., Chughtai M.I. (2021). The Role of Oxidative Stress and Antioxidant Balance in Pregnancy. Mediat. Inflamm..

[11] Zhang S., Wu Z., Heng J., Song H., Tian M., Chen F., Guan W. (2020). Combined Yeast Culture and Organic Selenium Supplementation during Late Gestation and Lactation Improve Preweaning Piglet Performance by Enhancing the Antioxidant Capacity and Milk Content in Nutrient-Restricted Sows. Anim. Nutr..

[12] Zheng S., Qin G., Zhen Y., Zhang X., Chen X., Dong J., Li C., Aschalew N.D., Wang T., Sun Z. (2021). Correlation of Oxidative Stress-Related Indicators with Milk Composition and Metabolites in Early Lactating Dairy Cows. Vet. Med. Sci..

[13] Sonnenburg J.L., Bäckhed F. (2016). Diet–Microbiota Interactions as Moderators of Human Metabolism. Nature.

[14] Odamaki T., Kato K., Sugahara H., Hashikura N., Takahashi S., Xiao J., Abe F., Osawa R. (2016). Age-Related Changes in Gut Microbiota Composition from Newborn to Centenarian: A Cross-Sectional Study. BMC. Microbiol..

[15] Hasan N., Yang H. (2019). Factors Affecting the Composition of the Gut Microbiota, and Its Modulation. PeerJ..

[16] Gorczyca K., Obuchowska A., Kimber-Trojnar Ż., Wierzchowska-Opoka M., Leszczyńska-Gorzelak B. (2022). Changes in the Gut Microbiome and Pathologies in Pregnancy. Int. J. Environ. Res. Public Health.

[17] Koren O., Goodrich J.K., Cullender T.C., Spor A., Laitinen K., Bäckhed H.K., Gonzalez A., Werner J.J., Angenent L.T., Knight R. (2012). Host Remodeling of the Gut Microbiome and Metabolic Changes during Pregnancy. Cell.

[18] Miko E., Csaszar A., Bodis J., Kovacs K. (2022). The Maternal–Fetal Gut Microbiota Axis: Physiological Changes, Dietary Influence, and Modulation Possibilities. Life.

[19] Butel M.-J., Waligora-Dupriet A.-J., Wydau-Dematteis S. (2018). The Developing Gut Microbiota and Its Consequences for Health. J. Dev. Orig. Health Dis..

[20] Lombrea A., Antal D., Ardelean F., Avram S., Pavel I.Z., Vlaia L., Mut A.-M., Diaconeasa Z., Dehelean C.A., Soica C. (2020). A Recent Insight Regarding the Phytochemistry and Bioactivity of *Origanum vulgare* L. Essential Oil. Int. J. Mol. Sci..

[21] Leyva-López N., Gutiérrez-Grijalva E.P., Vazquez-Olivo G., Heredia J.B. (2017). Essential Oils of Oregano: Biological Activity beyond Their Antimicrobial Properties. Molecules.

[22] Khafaga A.F., Naiel M.A.E., Dawood M.A.O., Abdel-Latif H.M.R. (2020). Dietary Origanum Vulgare Essential Oil Attenuates Cypermethrin-Induced Biochemical Changes, Oxidative Stress, Histopathological Alterations, Apoptosis, and Reduces DNA Damage in Common Carp (*Cyprinus carpio*). Aquat. Toxicol..

[23] Maral H., Ulupınar S., Baydır A.T., Özbay S., Altınkaynak K., Şebin E., Şiktar E., Kishalı N.F., Buzdağlı Y., Gençoğlu C. (2022). Effect of Origanum Dubium, Origanum Vulgare Subsp. Hirtum, and Lavandula Angustifolia Essential Oils on Lipid Profiles and Liver Biomarkers in Athletes. Z. Naturforsch. C J. Biosci..

[24] Serrano-Jara D., Rivera-Gomis J., Tornel J.A., Jordán M.J., Martínez-Conesa C., Pablo M.J.C. (2024). Oregano Essential Oil and Purple Garlic Powder Effects on Intestinal Health, Microbiota Indicators and Antimicrobial Resistance as Feed Additives in Weaning Piglets. Animals.

[25] Salehi B., Mishra A.P., Shukla I., Sharifi-Rad M., Contreras M.d.M., Segura-Carretero A., Fathi H., Nasrabadi N.N., Kobarfard F., Sharifi-Rad J. (2018). Thymol, Thyme, and Other Plant Sources: Health and Potential Uses. Phytother. Res..

[26] Silva E.R., de Carvalho F.O., Teixeira L.G.B., Santos N.G.L., Felipe F.A., Santana H.S.R., Shanmugam S., Júnior L.J.Q., de Souza Araújo A.A., Nunes P.S. (2018). Pharmacological Effects of Carvacrol in In Vitro Studies: A Review. Curr. Pharm. Des..

[27] Tan C., Wei H., Sun H., Ao J., Long G., Jiang S., Peng J. (2015). Effects of Dietary Supplementation of Oregano Essential Oil to Sows on Oxidative Stress Status, Lactation Feed Intake of Sows, and Piglet Performance. Biomed. Res. Int..

[28] Wang W., Wang Z., Ming D., Huang C., Xu S., Li Z., Wang Z., Liu H., Zeng X., Wang F. (2022). Effect of Maternal Dietary Starch-to-Fat Ratio and Daily Energy Intake during Late Pregnancy on the Performance and Lipid Metabolism of Primiparous Sows and Newborn Piglets. J. Anim. Sci..

[29] Wang L., Hou Y., Yi D., Li Y., Ding B., Zhu H., Liu J., Xiao H., Wu G. (2015). Dietary Supplementation with Glutamate Precursor α-Ketoglutarate Attenuates Lipopolysaccharide-Induced Liver Injury in Young Pigs. Amino Acids.

[30] Chen W., Wang W., Zhou L., Zhou J., He L., Li J., Xu X., Wang J., Wang L. (2022). Elevated AST/ALT Ratio Is Associated with All-cause Mortality and Cancer Incident. J. Clin. Lab. Anal..

[31] Daoudi N.E., Bnouham M. (2020). Hepatoprotective Essential Oils: A Review. J. Pharmacopunct..

[32] Haque M.R., Ansari S.H., Najmi A.K., Ahmad M.A. (2014). Monoterpene Phenolic Compound Thymol Prevents High Fat Diet Induced Obesity in Murine Model. Toxicol. Mech. Method..

[33] Mauri M., Calmarza P., Ibarretxe D. (2021). Dislipemias y Embarazo, Una Puesta al Día. Clin. Investig. Arterioscler..

[34] Schneider I., Kressel G., Meyer A., Krings U., Berger R.G., Hahn A. (2011). Lipid Lowering Effects of Oyster Mushroom (*Pleurotus ostreatus)* in Humans. J. Funct. Foods.

[35] Saravanan S., Pari L. (2015). Role of Thymol on Hyperglycemia and Hyperlipidemia in High Fat Diet-Induced Type 2 Diabetic C57BL/6J Mice. Eur. J. Pharmacol..

[36] Aristatile B., Al-Numair K.S., Veeramania C., Pugalendi K.V. (2009). Antihyperlipidemic Effect of Carvacrol on D-Galactosamine Induced Hepatotoxic Rats. J. Basic Clin. Physiol. Pharmacol..

[37] Basmacioğlu Malayoğlu H., Baysal Ş., Misirlioğlu Z., Polat M., Yilmaz H., Turan N. (2010). Effects of Oregano Essential Oil with or without Feed Enzymes on Growth Performance, Digestive Enzyme, Nutrient Digestibility, Lipid Metabolism and Immune Response of Broilers Fed on Wheat–Soybean Meal Diets. Br. Poultry Sci..

[38] Costa H.A., Dias C.J.M., Martins V.d.A., de Araujo S.A., da Silva D.P., Mendes V.S., de Oliveira M.N.S., Mostarda C.T., Borges A.C.R., Ribeiro R.M. (2021). Effect of Treatment with Carvacrol and Aerobic Training on Cardiovascular Function in Spontaneously Hypertensive Rats. Exp. Physiol..

[39] Kosmas C.E., Rodriguez Polanco S., Bousvarou M.D., Papakonstantinou E.J., Peña Genao E., Guzman E., Kostara C.E. (2023). The Triglyceride/High-Density Lipoprotein Cholesterol (TG/HDL-C) Ratio as a Risk Marker for Metabolic Syndrome and Cardiovascular Disease. Diagnostics.

[40] Cheng C., Wei H., Yu H., Xu C., Jiang S., Peng J. (2018). Metabolic Syndrome During Perinatal Period in Sows and the Link With Gut Microbiota and Metabolites. Front. Microbiol..

[41] Tan C., Wei H., Ao J., Long G., Peng J. (2016). Inclusion of Konjac Flour in the Gestation Diet Changes the Gut Microbiota, Alleviates Oxidative Stress, and Improves Insulin Sensitivity in Sows. Appl. Environ. Microbiol..

[42] Tsikas D. (2017). Assessment of Lipid Peroxidation by Measuring Malondialdehyde (MDA) and Relatives in Biological Samples: Analytical and Biological Challenges. Anal. Biochem..

[43] Llana-Ruiz-Cabello M., Gutiérrez-Praena D., Puerto M., Pichardo S., Jos Á., Cameán A.M. (2015). In Vitro Pro-Oxidant/Antioxidant Role of Carvacrol, Thymol and Their Mixture in the Intestinal Caco-2 Cell Line. Toxicol. In Vitro.

[44] El-Sayed E.M., Abd-Allah A.R., Mansour A.M., EL-Arabey A.A. (2015). Thymol and Carvacrol Prevent Cisplatin-Induced Nephrotoxicity by Abrogation of Oxidative Stress, Inflammation, and Apoptosis in Rats. J. Biochem. Mol. Toxicol..

[45] Zou Y., Wang J., Peng J., Wei H. (2016). Oregano Essential Oil Induces SOD1 and GSH Expression through Nrf2 Activation and Alleviates Hydrogen Peroxide-Induced Oxidative Damage in IPEC-J2 Cells. Oxid. Med. Cell. Longev..

[46] Shin N.-R., Whon T.W., Bae J.-W. (2015). Proteobacteria: Microbial Signature of Dysbiosis in Gut Microbiota. Trends. Biotechnol..

[47] Ismail H.T.H. (2022). The Ameliorative Efficacy of Thymus Vulgaris Essential Oil against Escherichia Coli O157:H7-Induced Hematological Alterations, Hepatorenal Dysfunction and Immune-Inflammatory Disturbances in Experimentally Infected Rats. Environ. Sci. Pollut. Res..

[48] Satora M., Magdziarz M., Rząsa A., Rypuła K., Płoneczka-Janeczko K. (2020). Insight into the Intestinal Microbiome of Farrowing Sows Following the Administration of Garlic (*Allium sativum*) Extract and Probiotic Bacteria Cultures under Farming Conditions. BMC. Vet. Res..

[49] Andrade-Ochoa S., Chacón-Vargas K.F., Sánchez-Torres L.E., Rivera-Chavira B.E., Nogueda-Torres B., Nevárez-Moorillón G.V. (2021). Differential Antimicrobial Effect of Essential Oils and Their Main Components: Insights Based on the Cell Membrane and External Structure. Membranes.

[50] Van den Abbeele P., Belzer C., Goossens M., Kleerebezem M., De Vos W.M., Thas O., De Weirdt R., Kerckhof F.-M., Van de Wiele T. (2013). Butyrate-Producing Clostridium Cluster XIVa Species Specifically Colonize Mucins in an in vitro Gut Model. ISME J..

[51] Tett A., Pasolli E., Masetti G., Ercolini D., Segata N. (2021). Prevotella Diversity, Niches and Interactions with the Human Host. Nat. Rev. Microbiol..

[52] Szajewska H., Kołodziej M. (2015). Systematic Review with Meta-Analysis: Lactobacillus Rhamnosus GG in the Prevention of Antibiotic-Associated Diarrhoea in Children and Adults. Aliment. Pharmacol. Ther..

[53] Yadav A.K., Tyagi A., Kumar A., Panwar S., Grover S., Saklani A.C., Hemalatha R., Batish V.K. (2017). Adhesion of Lactobacilli and Their Anti-Infectivity Potential. Crit. Rev. Food. Sci. Nutr..

[54] Betancur C., Martínez Y., Tellez-Isaias G., Castillo R., Ding X. (2021). Effect of Oral Administration with Lactobacillus Plantarum CAM6 Strain on Sows during Gestation-Lactation and the Derived Impact on Their Progeny Performance. Mediat. Inflamm..

